# Educational Nutrition Workshops: Impact on Nutritional Status in Organized Living Beneficiaries with Severe Mental Disorders and Their Caregivers

**DOI:** 10.3390/nu16223922

**Published:** 2024-11-17

**Authors:** Lidija Šoher, Milica Cvijetić Stokanović, Sara Prša, Daniela Čačić Kenjerić

**Affiliations:** 1 Department of Food and Nutrition Research, Faculty of Food Technology Osijek, Josip Juraj Strossmayer University of Osijek, Franje Kuhača 18, 31000 Osijek, Croatia; lidija.soher@ptfos.hr (L.Š.); mcvijetic@ptfos.hr (M.C.S.); 2 Center for Providing Community Services Osijek “ME Just Like YOU”, Martina Divalta 2, 31000 Osijek, Croatia; sara.prsa10@gmail.com

**Keywords:** intervention, educational workshops, severe mental disorders, caregivers, organized living

## Abstract

Background/Objectives: Individuals with severe mental disorders often face challenges in maintaining a healthy lifestyle, including proper dietary habits. Educational nutrition workshops, as a form of nutritional intervention, may play an important role in improving their nutritional status. This study aims to assess the impact of nutritional workshops on the dietary habits and nutritional status of individuals with severe mental disorders and their caregivers. Methods: This study involved 65 participants, namely 46 individuals with mental disorders living in organized settings (beneficiaries) and 19 caregivers. The nutritional intervention consisted of two cycles of workshops, encompassing a total of four educational workshops. Results: Results before and after the intervention showed positive changes in nutritional status and dietary intake. Beneficiaries lost on average 3.5 kg of body weight, while body fat decreased by 3.5% and visceral fat by 1.9 points. In the group of caregivers, body fat decreased by 3.2%. Energy intake (*p* < 0.05), total fat (*p* < 0.01), saturated fatty acid (*p* < 0.05), polyunsaturated fatty acid (*p* < 0.05), and sodium (*p* < 0.05) intake decreased in beneficiaries compared to intake before workshops, while dietary fiber (*p* < 0.05), vitamin C (*p* < 0.05), and fruit (*p* < 0.01) intake increased. In caregivers, the decrease in carbohydrate (*p* < 0.05), total sugar (*p* < 0.01), and dietary fiber (*p* < 0.05) intake was recorded. Conclusion: This study provides a strong foundation for future research and the implementation of educational programs as part of comprehensive care for individuals with severe mental disorders.

## 1. Introduction

Mental health is an integral part of overall health and represents more than mere absence of mental disorders. In 2019, it was estimated that one out of eight people in the world lives with some form of mental disorder. When it comes to mental health, most people do not have access to effective care or, due to the stigma associated with mental health, do not seek help. According to WHO data, the prevalence of mental disorders is 13.4% [1]. Mental challenges or disorders encompass a range of symptoms and impairments, characterized by psychological, biological, and social dysfunction [1]. Treatment requires a multidisciplinary approach, a combination of pharmacology and psychosocial therapy. Today, the basis of the treatment in clinical practice is the use of antipsychotics [2]. Antipsychotics, particularly atypical antipsychotics, are associated with dyslipidemia, hypertension, hyperglycemia, an increase in body weight and waist circumference, a high percentage of adipose tissue, constipation, and other side effects [3,4,5].

Overweight and obesity present a serious problem among people with severe mental disorders, including schizophrenia, schizotypal disorders, bipolar disorder, and depressive disorders [1]. The prevalence of overweight and obesity ranges from 10% to 60% in people with severe mental disorders [3,6]. In addition to an elevated body mass index (BMI), individuals with severe mental disorders have an increased level of body fat and reduced muscle mass [5,7]. Certain population characteristics have been identified as factors influencing nutritional status and related comorbidities: socioeconomic factors [8], tobacco use [9,10], unhealthy dietary patterns [11], low physical activity [12], and pharmacological therapy [4,13]. The unhealthy dietary patterns of this population are reflected in a high intake of fats, saturated fatty acids, sugars, and alcohol, alongside a reduced intake of dietary fiber, fruits, vegetables, and fish [9,10,11].

Various institutions such as social welfare institutions, employment services, and other non-governmental organizations, in addition to health institutions, are often involved in the treatment of people with severe mental disorders [14]. Community services usually include day-care and rehabilitation centers, a mobile crisis intervention team, employment assistance, organized housing service, and other psychosocial treatments aimed at training people for independent living, work, and improving patient’s life quality [14]. In providing the service of organized housing and assistance in everyday activities, educational nutritional workshops could be of great use. Nutrition education encourages individuals to learn and apply healthy eating habits in everyday life, by providing information, tools, and resources that support the decision-making process and behavior change [15]. Nutritional interventions are low-risk treatments, do not require significant investments, and have great potential [16]. Programs often consist of educational nutrition workshops, learning how to read declarations, cooking classes, household budget planning, and grocery shopping [15,16]. Intervention studies have confirmed the feasibility and acceptability of dietary interventions in populations with mental disorders [4,16,17,18,19]. These interventions can lead to subsequent weight reduction and decreased cardiovascular risk [3,4,20]. Studies are often heterogeneous in terms of diagnosed mental disorders, types of dietary interventions, and assessed outcomes, which makes comparison and generalization of the results difficult.

This study aimed to assess whether a series of educational nutrition workshops have an impact on the dietary habits and nutritional status of individuals with severe mental disorders and their caregivers.

## 2. Materials and Methods

This prospective study with elements of community-based intervention was conducted at the Department of Food and Nutrition Research, Faculty of Food Technology Osijek, and Center for Providing Community Services Osijek, “ME just like YOU”, from October 2021 to July 2022. A cooperation agreement was signed between the faculty and the center (class: 406-07/23-01/01; reg. no.: 2158-82-01-23-29), and concept and methodology was approved by the Ethics Committee for Human Research of the Faculty of Food Technology Osijek on July 23, 2021 (class: 033-08/21-01/01; reg. no.: 2158-82-01-21-58). The center is a social institution primarily focused on integrating individuals with mental disorders into the community. It provides a wide range of services, including organized living with four levels of care: occasional, daily short-term care, daily intensive care, and comprehensive care. Parts of the study, including participant recruitment, administration of the questionnaires, sociodemographic characteristics, and anthropometric and dietary assessments have been described elsewhere [21].

### 2.1. Study Design

**Figure 1:**
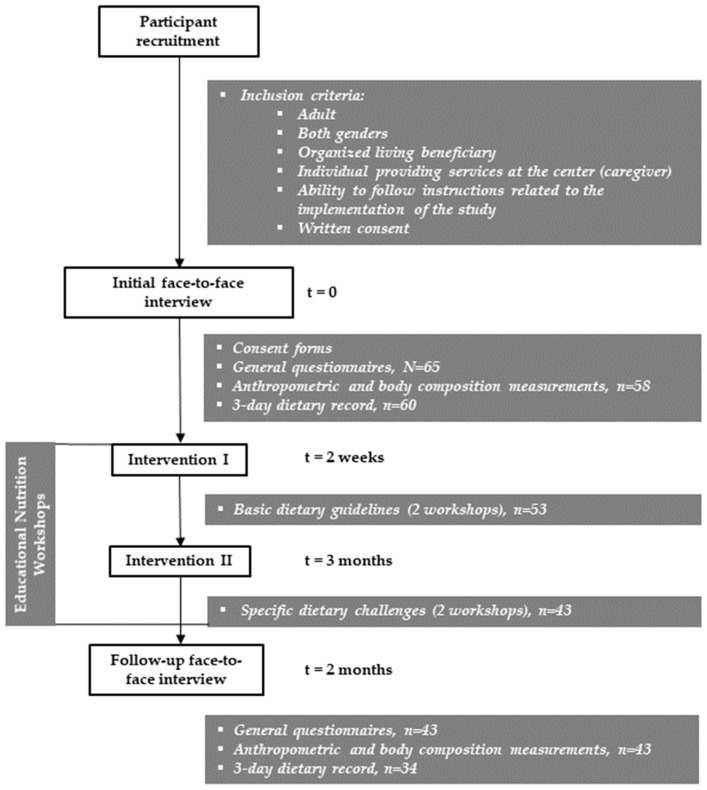
Study design flow chart.

This study consisted, after participant recruitment, of initial face-to-face interviews and measurements, interventions in the form of educational nutritional workshops, and follow-up interviews (Figure 1).

The initial face-to-face interviews were carried out over 15 appointments (~2 h) with smaller groups of participants (~4 participants). The participants were divided into groups taking into account the epidemiological measures (COVID-19), the daily schedule of the beneficiaries, and whether they live in the same organized living unit. During the initial face-to-face interview, participants signed consent forms and questionnaires developed specifically for this study were filled out. In collaboration with the center’s psychologists, all questionnaires were developed and adapted to the population for which they were intended. Anthropometric measurements and body composition assessments were also conducted during this time. To assess dietary intake at the start of the study, participants were given detailed instructions on keeping a 3-day dietary record (3-dDR).

Based on the data gathered during the initial dietary assessment, educational nutritional workshops were designed. The planned interval between the two cycles of workshops was two weeks. Due to the epidemiological measures (COVID-19) modified working conditions of the center and the inability to organize workshops, the interval between the workshop cycles was extended to three months.

After the intervention, the impact of the educational workshops on dietary habits and nutritional status was assessed by two-month follow-up interviews and measurements of all studied parameters.

### 2.2. Study Population

At the time of the study, 97 beneficiaries of organized living were living in approximately 30 housing units in the city of Osijek. The inclusion criteria for the study were as follows: adults, both genders, organized living beneficiaries, and individuals providing services at the center (caregivers) who could provide written consent and were able to follow instructions related to the implementation of the study. All beneficiaries and caregivers who met the inclusion criteria were given equal opportunity to participate. Participation in the study was voluntary. A total of 65 participants were included in the study, 46 beneficiaries (Table 1) and 19 caregivers. Caregivers were individuals employed at the center in the position of caregiver/assistant in organized living unit. Each caregiver was in daily contact with primarily the same housing unit and participated in certain aspects of life within the unit. The term “participants” in this study encompasses both beneficiaries and caregivers. The study encompassed three levels of care: daily short-term care (2.5 h/day), daily intensive care (16 h/day), and comprehensive care (24 h/day).

### 2.3. Nutritional Status and Dietary Intake Assessment

Two methods were used to determine nutritional status: BMI and body composition. Body weight (kg) and body composition (body fat, muscle mass, and visceral fat level) were measured using an Omron BF500 analyzer (Omron Healthcare Co., Ltd., Muko, Japan). Body weight was measured without shoes, at room temperature, while wearing clothes with an accuracy of 0.1 kg. The results were not further adjusted for clothing. Participants positioned themselves at the center of the scale platform, ensuring that their body weight was evenly balanced on both feet [22]. The measurement of body composition was performed according to the manufacturer’s instructions [23]. Body height (cm) was measured using a portable anthropometer (Seca 213, Hamburg, Germany), with the participant standing upright on a flat surface without shoes. When possible, the participant’s head was positioned in the Frankfurt plane. The headpiece was placed onto the participant’s head, and the height was recorded to the nearest 0.5 cm [22]. Body composition data were collected for 39 users. In certain instances, measurements could not be performed due to pronounced tremors or other disturbances present at the time measurement took place.

To assess dietary intake 3-dDR was used. A three-day period was chosen, two weekdays and one weekend day, to avoid overburdening participants. Standard household measures (e.g., tablespoon, teaspoon, cup, glass, plate), food portions, pieces (e.g., a slice of bread), or packaging sizes were used to estimate portion sizes. In addition to household measures, a photographic atlas featuring various portion sizes of typical national dishes was used [24]. The use of dietary supplements was also recorded in the food diary. Upon completion, the food diaries were submitted to the main researcher. The diaries were thoroughly reviewed as soon as possible to minimize potential oversights. Since caregivers participated in purchasing, preparing, and serving meals, they were used as surrogate sources of information during the 3-dDR review. The collected data were processed on an individual basis using the computer application Program Prehrane 6.41.0 (IG PROG, Rijeka, Croatia).

### 2.4. Educational Nutrition Workshops

The intervention consisted of two cycles. The first cycle (Intervention I) covered basic dietary guidelines and consisted of two workshops: “Food Groups and Nutrient Sources” and “Let’s make a Healthy Eating Plate”! The second cycle (Intervention II) focused on specific dietary challenges identified through initial dietary assessment. A total of 20% of organized living units had fresh fruit on their daily menu, 10% had fresh vegetables (salads), and 20% had cooked vegetables (side dish or stew). In addition, 43% of total energy intake came from fat. Based on this, the second cycle workshops were designed: “How to eat more colors?” and “Fat Sources in our Diet”.

All workshops were developed in collaboration with center’s psychologist. This enabled the creation of education aids and assignments tailored to the workshop’s objective and participants’ capability. Due to the pandemic and the direct contact and interactive approach of the workshops, workshops were held in smaller groups. The groups consisted of 10 participants (beneficiaries and caregivers). Both cycles of workshops (4 workshops) were delivered over 6 sessions, each lasting 60 min. The workshop protocols described in Table 2 were developed to ensure a consistent approach and execution of the workshops across all sessions.

### 2.5. Data Analysis

The data were organized into a database as categorical (participant group) and continuous (interval) variables (weight, BMI, body fat, muscle mass, visceral fat rating, energy intake, and intake of 30 selected macro- and micronutrients). The complete results of macro- and micronutrient intake are available in the supplementary table (Table S1). Standard descriptive statistics were used to describe the results. Mean ($\bar{x}$) and standard deviation (SD), were used to describe continuous (interval) data. 

The normality of the distribution was tested using the Shapiro–Wilk test. Most variables did not show normal distribution, and non-parametric tests were used. The impact of the intervention was analyzed using per-protocol analysis. Differences between two dependent variables, before and after the intervention, were tested using Wilcoxon matched pairs test. The level of significance was set at *p* < 0.05. Data analysis was conducted using the statistical software package Statistica version 14.0.1.25 (1984–2020 TIBCO Software Inc., Hamburg, Germany) and Microsoft Excel 2016 (version 16.0.5413.1000, 2016 Microsoft Corporation, Redmond, WA, USA).

## 3. Results and Discussion

Dietary interventions, which often involve a combination of nutrition education and physical activity plans, may significantly improve anthropometric characteristics, disease symptoms, and overall functioning and quality of life in individuals with severe mental disorders [4,16,17,18,19]. The timeframe in which a measurable change is expected may depend on the type of intervention, the population included in the study, and many other factors [25]. Depending on the intervention, changes in dietary intake can be observed in a shorter time, while changes in nutritional status parameters may be observed in a few months. In total, 65 participants were included in the study, namely 46 beneficiaries (43% male) and 19 caregivers (all female). A total of 80.3% of beneficiaries and 61.9% of caregivers were overweight or obese. A total of 80% of beneficiaries were diagnosed with schizophrenia with 54.3% of beneficiaries in intensive daily care [21].

### 3.1. Educational Nutrition Workshops Impact on Nutritional Status

Differences in anthropometric characteristics and body composition before and after intervention are shown in Table 3, for both caregivers and beneficiaries. Significant differences were found for body fat (*p* = 0.009) and muscle mass (*p* = 0.013) in caregivers. On average, body fat decreased by 3.2%, and muscle mass increased by 1.7% compared to baseline. Although not significant, changes in body weight, BMI, and visceral fat level were observed. In four caregivers, a decrease in visceral fat (−1) was recorded. Similarly to our results, Rossimel et al. [26] found no significant impact of nutritional counseling on weight and BMI in healthcare professionals working with patients with severe mental disorders [26]. In contrast, the difference was observed for all parameters in the beneficiary group: weight (*p* = 0.008), BMI (*p* = 0.016), body fat (*p* = 0.003), muscle mass (*p* = 0.007), and visceral fat (*p* = 0.009). Beneficiaries who lost weight (67.7%) lost on average 3.5 kg, while for those who gained weight, an average increase of 2.1 kg was observed. Body and visceral fat decreased on average by 3.5% and 1.9, respectively, while muscle mass increased by 1.6% in the majority of beneficiaries. Weight loss or minimal weight gain after nutritional intervention are previously reported in several studies [25,27,28,29]. Bartels et al. [27] reported an average weight loss of 2.09 kg after intervention, while Brown et al. [25] reported an average weight loss of 2.18 kg three months after intervention. In the current study, the average weight loss in the beneficiary group exceeds the averages reported in previous studies. This trend is also evident in other indicators. When it comes to BMI, after a six-month intervention, Magni et al. [30] recorded an average BMI reduction of 0.6 kg/m^2^ in individuals with mental disorders, while Gallagher et al. [20] found no significant reduction [20,30]. Our results show a somewhat higher average BMI reduction of 1.3 kg/m^2^ in 67.7% of beneficiaries. Variations in the results across studies may be due to different methodologies, study designs, interventions, sample sizes, and characteristics of the studied population.

### 3.2. Educational Nutrition Workshops Impact on Dietary Intake

Differences in dietary intake before and after intervention are presented in Table 4. Significant differences in the intake before and after the intervention in the caregivers’ group were observed for carbohydrates (*p* = 0.021), total sugars (*p* = 0.008), dietary fiber (*p* = 0.038), and chloride (*p* = 0.038). In the beneficiary group, significant differences were found in energy intake (*p* = 0.035), fiber (*p* = 0.042), total fat (*p* = 0.006), saturated fatty acids (*p* = 0.013), polyunsaturated fatty acids (*p* = 0.028), sodium (*p* = 0.042), vitamin C (*p* = 0.030), and fruit intake (*p* = 0.005).

A reduction of 429.1 kcal/day was noted in beneficiaries. The average intake before intervention was 1850.0 ± 444.4 kcal/day, and after the intervention, it was 1641.7 ± 279.5 kcal/day. Of the 25 participants in this group, 17 had a lower energy intake. On the other hand, the increase was, on average, 237.8 kcal. A similar reduction in energy intake, averaging 468 kcal, was noted in the study by Teasdale et al. [31], which aligns with these results. A slightly higher reduction in caloric intake was recorded in the study which included individuals with mental disorders by Curtis et al. [29], where the average energy intake decreased by 507.9 kcal/day after the intervention [29]. An appropriate reduction in energy intake can be a useful mechanism for weight loss since weight gain is often a side effect of antipsychotics use. The majority of beneficiaries (76.2%) failed to meet daily energy needs in this study, highlighting the need for a comprehensive approach to dietary habits assessment in this population [21,31]. Under-reporting and, consequently, lower energy and nutrient intake, among other things, may be due to cognitive and motivational challenges in people with severe mental disorders [10,21]. A significant change was noticed in carbohydrate and total sugar intake within the caregiver group, with average changes of −47.9 g/day and −26.7 g/day, respectively. In both the caregiver and beneficiary groups, changes in average dietary fiber intake were recorded. Among caregivers, average intake decreased from 14.5 ± 2.8 g/day to 11.2 ± 2.4 g/day, while in the beneficiary group, it increased from 10.6 ± 4.1 g/day to 12.6 ± 3.6 g/day. Despite the increase, dietary fiber intake remained below the recommendations for adults (25 g/day) [32]. The low dietary fiber intake in people with severe mental disorders has been mentioned in previous studies [33]. Given the known positive effect of fiber on constipation, a frequent side effect of antipsychotic therapy, achieving adequate dietary fiber intake in this population is important [34,35].

Frequent high fat intake, characterized by high intake of saturated fat and low intake of polyunsaturated fatty acids, has been previously reported in people with mental disorders [10,11,21,33,36]. After the nutrition intervention in our study, fat intake changed in the beneficiary group (72%). On average, fat intake was 30.0 g/day lower compared to intake before intervention. The reduction in fat intake observed in this study is higher than 22 g/day reported by Teasdale et al. [31]. Compared to intake before the intervention, the intake of saturated fat decreased in most beneficiaries (−13.3 g/day; 72%), but there was also a decrease in polyunsaturated fatty acid intake (−13.5 g/day; 60%). Given the proven positive effects of polyunsaturated fatty acids and an adequate ratio of fatty acids, which may benefit a wide range of psychiatric, neurological, and developmental disorders in adults, finding successful strategies to increase their intake could be beneficial [37]. The often high cost of foods rich in polyunsaturated fatty acids could pose a problem for low-income populations like this one. On the other hand, the demonstrated reduction in fat and saturated fat intake could potentially lower health risks in this population [3].

A lower intake of fruits and vegetables has been observed among individuals with mental disorders. In this study, conducted by Heald et al. [9], only 13.5% of participants with schizophrenia consumed the recommended five or more servings of fruits and vegetables daily. The average daily intake of fruit was 1.1 ± 1.0 servings, while the average intake of vegetables was 1.7 ± 1.2 servings [9], which is not in accordance with presented results. Before the intervention, beneficiaries had a daily intake of fruit of 0.51 ± 0.8 servings, and 0.94 ± 0.4 servings of vegetables. After the intervention, a significant difference was observed only for fruit intake in the beneficiary group, with an average intake after the intervention of 1.67 ± 1.2 servings. Significant changes in fruit intake have also been observed in other studies that included interventions in the form of nutritional education [38,39]. Interventions aimed at improving dietary quality, such as increasing the consumption of fruits and vegetables while reducing the intake of nutritionally poor foods—such as processed foods high in calories, saturated fats, added sugars, and salt—may be crucial for enhancing the physical health of individuals with severe mental disorders. Increasing the intake of fruits and vegetables can provide essential vitamins, minerals, and antioxidants, while also promoting satiety with a lower caloric intake [31].

A comparison of vitamin and mineral intake before and after the intervention revealed significant differences in chloride intake in the caregiver group and sodium and vitamin C intake in the beneficiary group. Sodium intake changed in 64% of beneficiaries compared to intake before the intervention, with an average decrease of 1.1 g/day. Only one participant in the beneficiary group met the recommended sodium intake (2 g/day) after the intervention. Other studies have previously reported on the reduction in sodium intake following the nutritional intervention, which may play a key role in preventing cardiovascular disease in this population [31]. The mean vitamin C intake before the intervention was 54.2 ± 32.9 mg/day, with a mean intake of 71.9 ± 26.1 mg/day after the intervention. Vitamin C intake increased in 68% of beneficiaries, with a mean increase of 37.3 mg/day. Although still below recommendations, these results are a step in the right direction, considering the lower diet quality often seen in people with mental disorders [40]. Cooking workshops conducted as part of dietary interventions in the study by Clark et al. [38] led to changes in calcium and vitamin D intake. A comparison of intake before and after the intervention revealed an increase in calcium and vitamin D intake in individuals with mental disorders [38]. Although not statistically significant, in the current study, calcium intake decreased, while vitamin D intake increased compared to intake before intervention. In addition to vitamin D [41], other vitamins and minerals, which have been proven to influence certain aspects of mental disorders, such as folate [42], vitamin B12 [43], B6, zinc [44], and magnesium, showed non-significant differences in intake after the intervention compared to intake before. Of the above-mentioned nutrients, only folate intake increased compared to intake before intervention (Table S1).

The intervention, in the form of educational nutrition workshops, resulted in changes in both nutritional status and dietary intake. Although all significant changes are in a positive direction, several limiting factors should be considered when interpreting the results. Challenges in conducting research involving people with severe mental disorders have been highlighted multiple times [10,11,28,45]. In the current study, selecting an appropriate dietary assessment method, recruiting participants and the lack of motivation among users and caregivers proved to be challenging. The study’s design, sample size, and absence of a control group may reduce the reliability of the collected data. The nature of the illness and the variability in cognitive abilities and motivation among organized living beneficiaries made it impossible to form a control group, which would have allowed for a clearer conclusion regarding the intervention’s impact. Therefore, the opportunity to participate in educational nutrition workshops was given to all interested beneficiaries. A unique dimension of this study was the involvement of caregivers, which allowed for more detailed and accurate data collection on the diet and lifestyle habits of beneficiaries, while also assessing the nutritional status of those in close proximity. Although more changes were expected in the caregiver’s group, a possible reason for their absence may be that their primary focus was on beneficiaries, during and after the workshops. Given the scarcity of literature on the nutrition of individuals with mental disorders in Croatia, this study, despite its limitations, makes a significant contribution to the field of care for people with mental disorders.

## 4. Conclusions

This prospective study with elements of community-based intervention involving both organized living beneficiaries with mental disorders and their caregivers, demonstrated the potential of educational nutrition workshops as an interventional tool. Comparing the values of observed parameters before and after the intervention, a positive impact on the nutritional status of participants was observed. The educational nutrition workshops demonstrated an impact on beneficiaries’ nutritional status and dietary intake, supporting the further research and development of accessible, low-risk tools in the care for people with mental disorders.

## Figures and Tables

**Table 1:** Number (%) of organized living beneficiaries included in the study.

Level of Care	Number of Organized Living Beneficiaries	Number (%) of Beneficiaries Included in the Study
Occasional	10	0 (0)
Daily short-term care	13	9 (69%)
Daily intensive care	56	25 (45%)
Comprehensive care	18	12 (67%)
Total	97	46 (47)

**Table 2:** Workshop protocols.

	Aim	Assignment	Educational Aid	Note
Intervention I				
Food groups and nutrient sources	Introduce participants to the concept of food groups and different nutrient sources in the diet.	Sort all food photographs into appropriate food groups (baskets).	126 cards with photographs of everyday food items 4 different size baskets with food group label	Food not commonly found (exotic fruit, etc.) on the menus was excluded to avoid potential negative impression regarding participants’ diet and socioeconomic status. Different basket sizes represented the recommended proportion of food groups in the diet.
Let’s make a healthy eating plate!	Convey the information about basic dietary guidelines through a healthy plate.	Arrange the food items on a paper plate according to the healthy plate guidelines.	Healthy plate picture Promotional catalogs from different retail chains Paper plates and cups Scissors and glue	Promotional catalogs are part of the planning of weekly menus in organized living units and, therefore, were used to link food selection and guidelines. Created plates served as reminders and encouragement for planning healthier meals after the workshops.
Intervention II				
How to eat more colors?	Teach participants about the importance of consuming fruits and vegetables and how to incorporate fruit and vegetables into their diet.	Using existing dishes and colorful fruits and vegetables to create a puzzle-like new daily menu.	Printed fruit and vegetable pictures 115 dishes from existing menus Crayons and markers Presentation with ideas on how to incorporate more fruit and vegetables into the diet	Not to impact menu costs, dishes, ingredients, and recipes already present on the menu were used.
Fat sources in our diet	Identify fat sources in the diet and instruct on recommended portions.	Using pictures of 11 food items assignment was to find food items in a word search puzzle and identify fat sources in the diet. Take part in a quiz about portion sizes.	Word search puzzle Quiz Crayons and markers Printed answers for the quiz	Participants responded positively to tasks that involved their creative expression. Therefore, they were required to color their answers for the quiz.

**Table 3:** Mean anthropometric and body composition parameters before and after intervention, and the mean difference in parameters determined before and after the intervention.

	n*	Before Intervention $\bar{x}$ ± SD	After Intervention $\bar{x}$ ± SD	*p* **	↓ After Intervention *n* (%) ***	↓ After Intervention $\bar{x}$	↑ After Intervention *n* (%)	↑ After Intervention $\bar{x}$
Caregivers (*n* = 12)								
Weight (kg)	12	75.9 ± 13.4	74.8 ± 13.2	0.071	10 (83.3)	−2.1	2 (16.7)	2.9
BMI (kg/m^2^)	12	27.8 ± 5.6	26.8 ± 5.6	0.078	10 (83.3)	−0.7	2 (16.7)	1.1
Body fat (%)	11	38.3 ± 8.0	35.6 ± 8.5	**0.009**	10 (91.0)	−3.2	1 (9.0)	2.2
Muscle mass (%)	11	26.6 ± 3.1	28.1 ± 3.4	**0.013**	1 (9.0)	−1.6	10 (91.0)	1.7
Visceral fat rating	4	7.7 ± 2.9	7.4 ± 2.7	0.068	4 (100)	−1.0	-	-
Beneficiaries (*n* = 31)								
Weight (kg)	31	86.6 ± 16.1	84.9 ± 15.6	**0.008**	21 (67.7)	−3.5	10 (32.3)	2.1
BMI (kg/m^2^)	31	31.0 ± 6.1	30.3 ± 5.9	**0.016**	21 (67.7)	−1.3	10 (32.3)	0.8
Body fat (%)	29	39.1 ± 12.1	37.2 ± 12.4	**0.003**	20 (6.9)	−3.5	9 (31.1)	1.4
Muscle mass (%)	29	27.1 ± 6.0	28.0 ± 6.1	**0.007**	7 (24.1)	−1.2	22 (75.9)	1.6
Visceral fat rating	19	10.6 ± 5.2	9.5 ± 5.0	**0.009**	14 (73.7)	−1.9	5 (26.3)	1.0

$\bar{x}$ ± SD—mean ± standard deviation; n*—participants in whom changes were observed; ** Wilcoxon matched pair test; *** change in parameters after intervention (↓ decrease, ↑ increase); statistical significance (*p* < 0.05) in bold.

**Table 4:** Mean dietary intake before and after intervention, and mean difference in parameters for whom significant difference was observed.

	n*	Before Intervention $\bar{x}$ ± SD	After Intervention $\bar{x}$ ± SD	*p* **	↓ After Intervention *n* (%) ***	↓ After Intervention $\bar{x}$	↑ After Intervention *n* (%)	↑ After Intervention $\bar{x}$
Caregivers (*n* = 9)								
Energy, kcal/day	9	1745.6 ± 218.5	1521.2 ± 448.7	0.086				
Protein, g/day	9	64.7 ± 20.1	58.9 ± 19.0	0.260				
Carbohydrates, g/day	9	213.6 ± 33.0	172.6 ± 45.8	**0.021**	8 (88.9)	−47.9	1 (11.1)	14.4
Total sugar, g/day	9	100.8 ± 26.3	74.2 ± 15.0	**0.008**	9 (100)	−26.7	-	-
Dietary fiber, g/day	9	14.5 ± 2.8	11.2 ± 2.4	**0.038**	8 (88.9)	−4.4	1 (11.1)	4.87
Total fat, g/day	9	73.3 ± 20.6	62.5 ± 29.8	0.109				
Saturated fat, g/day	9	25.6 ± 9.8	22.2 ± 11.9	0.374				
Polyunsaturated fatty acids, g/day	9	17.4 ± 5.8	18.3 ± 8.5	0.953				
Chloride, g/day	9	3.2 ± 1.3	3.0 ± 1.4	**0.038**	7 (77.8)	−1.1	2 (22.2)	0.5
Fruit, serving/day	9	1.9 ± 1.2	1.9 ± 1.0	0.767				
Vegetables, serving/day	9	2.0 ± 0.4	1.7 ± 1.2	0.214				
Beneficiaries (*n* = 25)								
Energy, kcal/day	25	1850.0 ± 444.4	1641.7 ± 279.5	**0.035**	17 (68.0)	−429.1	8 (32.0)	237.8
Protein, g/day	25	68.9 ± 15.8	66.1 ± 11.3	0.459				
Carbohydrates, g/day	25	200.8 ± 54.9	208.3 ± 85.6	0.638				
Total sugar, g/day	25	71.6 ± 41.1	71.2 ± 30.5	0.840				
Dietary fiber, g/day	25	10.6 ± 4.1	12.6 ± 3.6	**0.042**	7 (28.0)	−3.8	18 (72.0)	4.23
Total fat, g/day	25	88.9 ± 51.3	71.4 ± 16.1	**0.006**	18 (72.0)	−30.0	7 (28.0)	14.3
Saturated fat, g/day	25	32.8 ± 11.9	25.3 ± 7.1	**0.013**	18 (72.0)	−13.3	7 (28.0)	7.22
Polyunsaturated fatty acids, g/day	25	26.8 ± 8.3	20.3 ± 7.5	**0.028**	15 (60.0)	−13.5	10 (40.0)	5.15
Sodium, g/day	25	3.5 ± 9.7	3.0 ± 6.3	**0.042**	16 (64.0)	−1.1	9 (36.0)	0.5
Vitamin C, mg/day	25	54.2 ± 32.9	71.9 ± 26.1	**0.030**	8 (32.0)	−23.9	17 (68.0)	37.3
Fruit, serving/day	19	0.51 ± 0.8	1.67 ± 1.2	**0.005**	4 (21.1)	−1.0	15 (78.9)	1.73
Vegetables, serving/day	25	0.94 ± 0.4	1.41 ± 0.9	0.059				

$\bar{x}$ ± SD—mean ± standard deviation; n*—participants in whom changes were observed; ** Wilcoxon matched pair test; *** change in parameters after intervention (↓ decrease, ↑ increase); statistical significance (*p* < 0.05) in bold.

## Data Availability

The database is available at Josip Juraj Strossmayer University of Osijek, Faculty of Food Technology Osijek, Department of Food and Nutrition Research. The data are not publicly available due to privacy/legal/ethical reasons.

## Supplementary Materials

The following supporting information can be downloaded at: https://www.mdpi.com/article/10.3390/nu16223922/s1, Table S1: Mean energy, macro- and micronutrients intake before and after the intervention.
