# Supplementary material for: Educational Nutrition Workshops: Impact on Nutritional Status in Organized Living Beneficiaries with Severe Mental Disorders and Their Caregivers
Source: Nutrients. 2024 Nov 17;16(22):3922. doi: 10.3390/nu16223922 (PMC11597513; doi:10.3390/nu16223922)
Supplement: Supplementary file 1 [file nutrients-16-03922-s001.zip › nutrients-3304228-supplementary.pdf]

Table S1 Mean energy, macro- and micronutrients intake before and after the intervention

	Caregivers (n=9)				Beneficiaries (n=25)			
	n*	Before intervention x̄ ± SD	After intervention x̄ ± SD	p**	n*	Before intervention x̄ ± SD	After intervention x̄ ± SD	p**
Energy, kcal/day	9	1745.6 ± 218.5	1521.2 ± 448.7	0.086	25	1850.0 ± 444.4	1641.7 ± 279.5	**0.035**
Protein, g/day	9	64.7 ± 20.1	58.9 ± 19.0	0.260	25	68.9 ± 15.8	66.1 ± 11.3	0.459
Carbohydrates, g/day	9	213.6 ± 33.0	172.6 ± 45.8	**0.020**	25	200.8 ± 54.9	208.3 ± 85.6	0.638
Starch, g/day	9	91.61 ± 30.5	86.8 ± 39.0	0.441	25	120.7 ± 27.3	110.3 ± 21.8	0.128
Total sugars, g/day	9	100.8 ± 26.3	74.2 ± 15.0	**0.008**	25	71.6 ± 41.1	71.2 ± 30.5	0.840
Dietary fiber, g/day	9	14.5 ± 2.8	11.2 ± 2.4	**0.038**	25	10.6 ± 4.1	12.6 ± 3.6	**0.042**
Total fat, g/day	9	73.3 ± 20.6	62.5 ± 29.8	0.110	25	88.9 ± 51.3	71.4 ± 16.1	**0.006**
Saturated fat, g/day	9	25.6 ± 9.8	22.2 ± 11.9	0.374	25	32.8 ± 11.9	25.3 ± 7.1	**0.013**
Monounsaturated fatty acids, g/day	9	19.5 ± 9.9	14.7 ± 8.5	0.051	25	19.1 ± 5.9	18.6 ± 4.0	0.427
Polyunsaturated fatty acids, g/day	9	17.4 ± 5.8	18.3 ± 8.5	0.953	25	26.8 ± 8.3	20.3 ± 7.5	**0.028**
Trans fatty acids, g/day	9	0.8 ± 0.8	0.5 ± 9.3	0.374	25	0.6 ± 0.2	0.5 ± 0.4	0.109
Cholesterol, mg/day	9	228.8 ± 111.8	181.6 ± 112.6	0.441	25	263.1 ± 120.3	233.0 ± 84.8	0.510
Sodium, g/day	9	3.7 ± 1.0	2.9 ± 1.4	0.314	25	3.5 ± 9.7	3.0 ± 6.3	**0.042**
Potassium, mg/day	9	2473.8 ± 422.8	2396.7 ± 523.0	0.767	25	2240.7 ± 639.1	2423.1 ± 531.3	0.300
Calcium, mg/day	9	862.8 ± 264.9	785.9 ± 321.1	0.441	25	959.5 ± 294.1	884.6 ± 223.5	0.264
Chloride, g/day	9	3.21 ± 1.34	3.0 ± 1.4	**0.038**	25	3.6 ± 1.3	3.3 ± 0.7	0.276
Magnesium, mg/day	9	262.8 ± 143.4	213.6 ± 59.1	0.594	25	224.6 ± 68.7	233.0 ± 50.0	0.619
Phosphorus, mg/day	9	1081.7 ± 324.9	943.6 ± 341.2	0.314	25	1127.9 ± 273.3	1077.5 ± 206.5	0.427
Iron, mg/day	9	8.8 ± 1.6	8.1 ± 2.5	0.594	25	10.8 ± 3.8	10.2 ± 2.2	0.657
Copper, mg/day	9	0.9 ± 0.2	0.8 ± 0.3	0.374	25	1.1 ± 0.4	1.0 ± 0.2	0.581
Zinc, mg/day	9	6.7 ± 2.3	6.1 ± 2.5	0.260	25	7.6 ± 2.8	7.6 ± 1.7	0.830
Manganese, mg/day	9	1.9 ± 0.3	1.8 ± 0.7	0.374	25	2.0 ± 0.6	2.0 ± 0.4	0.872
Selenium, µg/day	9	30.6 ± 21.3	24.6 ± 14.8	0.374	25	26.2 ± 11.9	27.1 ± 10.2	0.619
Iodine, µg/day	9	53.7 ± 29.6	41.9 ± 21.4	0.173	25	45.3 ± 17.2	46.9 ± 16.3	0.638
Vitamin A, µg/day	9	655.7 ± 435.0	569.6 ± 447.0	0.594	25	508.5 ± 285.5	510.2 ± 217.2	0.798
Vitamin D, µg/day	9	2.1 ± 1.5	2.4 ± 3.3	0.953	25	3.4 ± 2.6	2.9 ± 2.7	0.339
Vitamin E, mg/day	9	11.0 ± 3.4	9.8 ± 2.6	0.441	25	8.7 ± 4.1	8.5 ± 2.8	0.872
Vitamin K, µg/day	9	133.7 ± 105.1	246.9 ± 440.1	0.767	25	65.1 ± 79.6	99.1 ± 126.9	0.545
Thiamine (B_1), mg/day	9	1.5 ± 0.6	1.2 ± 0.5	0.441	25	1.6 ± 0.5	1.5 ± 0.3	0.435
Riboflavin (B_2), mg/day	9	1.2 ± 0.5	1.6 ± 0.5	0.575	25	1.6 ± 0.6	1.5 ± 0.4	0.743

Niacin (B_3), mg/day	9	13.7 ± 4.8	13.5 ± 3.2	0.953	25	17.7 ± 5.9	16.0 ± 3.2	0.677
Pantothenic acid (B_5), mg/day	9	2.8 ± 1.3	2.4 ± 1.4	0.374	25	2.6 ± 1.5	2.9 ± 0.8	0.468
Pyridoxine (B_6), mg/day	9	1.9 ± 0.9	1.7 ± 0.6	0.767	25	1.8 ± 0.5	1.7 ± 0.5	0.936
Folate (B_9), μg/day	9	196.0 ± 53.8	192.8 ± 94.0	0.767	25	216.0 ± 64.3	233.2 ± 57.2	0.382
Cobalamin (B_{12}), mg/day	9	2.71 ± 2.4	2.6 ± 2.8	0.374	25	5.4 ± 4.9	4.6 ± 3.3	0.510
Vitamin C, mg/dan	9	113.6 ± 93.3	85.0 ± 42.4	0.441	25	54.2 ± 32.9	71.9 ± 26.1	**0.030**
Fruit, serving/day	9	1.9 ± 1.2	1.9 ± 1.0	0.767	19	0.51 ± 0.8	1.67 ± 1.2	**0.005**
Vegetables, serving/day	9	2.0 ± 0.4	1.7 ± 1.2	0.214	25	0.94 ± 0.4	1.41 ± 0.9	0.059

$\bar{x}$ ± SD - mean ± standard deviation; n* – participants in whom changes were observed; **Wilcoxon matched pair test; statistical significance ($p<0.05$) in bold
